# Toxicology

**Published:** 1837-04

**Authors:** 


### TOXICOLOGY.

*Case of Poisoning by Sulphuric Acid. Death after twelve weeks.* By Dr. Braun, of Fürth.

On the 12th of March, 1834, a young woman, just before going to bed, swallowed some liquid which had been prescribed for her by a person unknown, for an illness under which she was labouring. Immediately after having swallowed it, she perceived an extremely burning sensation in the mouth and throat. Not feeling alarmed, she went to bed; and on the next morning was found lying insensible, and unable to give any account of her condition. A quantity of mucus and blood was seen near the bed. About ten o'clock, she came to herself, but she was not brought to the house of her parents until the following day.

A portion of liquid which remained in the glass, out of which she had swallowed the dose, was examined on the 15th, and found to be sulphuric acid, though not in its highly concentrated state. The parents were informed of this, and the girl then for the first time confessed that she had taken the liquid as a medicine prescribed for her. Although three days had elapsed, she still complained of a violent burning sensation in the throat, with severe pain, and of almost an utter impossibility of swallowing. The tongue and throat appeared to be covered with a white incrustation. In the progress of time, these symptoms, under proper treatment, abated, and the power of swallowing returned. Water and milk were then freely prescribed, for the purpose of washing out the mouth and fauces; but the irritability of the stomach continued so great, that even at the end of the second week, if liquids or solids were taken in any quantity, they were immediately rejected. During the third week, the vomited matters resembled dark coffee-grounds in colour, and were highly offensive; but they afterwards assumed more of a mucous character. It was also observed, that after every meal, she suffered extreme pain in the region of the stomach, which did not abate until all that she had taken had been thrown off. Owing to this disturbance in the functions of the stomach, she became slowly more and more emaciated, and finally sank, apparently from pure exhaustion, on the 4th of June, *twelve weeks* after having swallowed the poison. Latterly, attempts were made to support her by nutritious enemata, but no means could arrest the progress of the fatal emaciation. During the whole period, her mind was calm, and her faculties clear.

An inspection of the body was made on the 7th of June, three days after death. It had wasted to a perfect skeleton: there were not the slightest vestiges of mammæ. It had undergone no putrefactive change. (N.B. Bodies which are emaciated, are, cæteris paribus, very slow in putrefying.) A small quantity of serous fluid was found in the chest, and about two ounces in the pericardium. With the exception of the heart being very flaccid, and the lungs presenting a few small tubercles, the thoracic viscera were healthy. The stomach was preternaturally large, and it contained a considerable quantity of greenish coloured offensive liquid. On the anterior wall of the stomach, and at the cardiac extremity, were two patches of ulceration, in which the mucous membrane had been entirely removed. There were no traces of ulceration in the intestinal canal; but here and there, the parietes were thinner than natural. Around the pylorus, the stomach was extremely thickened; the pylorus itself appeared perfectly cartilaginous, and the aperture was so *contracted* as only to admit the barrel of a quill. There was no other abnormal change. The reporter of the case feels satisfied that, had proper remedies, such as alkaline carbonates, been employed in the first instance, the fatal effects of the poison might have been prevented. Had the deceased not kept the circumstances concealed, it would not have been, he imagines, too late to have exhibited these remedies on the day following that on which she had taken the poison. It is certain that the acid must have been much diluted, when swallowed, or the fatal consequences would probably have been more speedily induced, and the morbid changes more extensive.

[*Remarks.* This case is not so well reported as the generality of those contained in Henke's Journal. In all cases of poisoning, it is important to know the probable quantity taken, as also, if it be a mineral acid, the probable degree of concentration in which it has been swallowed. The latter point, at least, might have been here determined from the residue of the acid found in the glass; and, indeed, with regard to sulphuric acid, the quantitative analysis is so simple, that, in general, if there be sufficient for its presence to be indicated, there is sufficient for its strength to be determined. We dwell upon this circumstance, because it is too much the custom, in the report of cases of poisoning by the mineral acids, to find it altogether neglected; and it requires but little reflection to show, that this question ought to be solved in order that one case should be fairly compared with another. This case presents, nevertheless, an interesting example of the secondarily fatal effects of the mineral acids, and furnishes additional evidence of the great length of time for which life may be protracted.

The morbid changes were entirely confined to the stomach, and were not very considerable; the ulceration was limited to a small portion of mucous membrane. The pylorus seems to have had its functions entirely destroyed, to have become indeed, almost impermeable to the passage of the food, a condition which at once accounts for the constant vomiting under which the deceased laboured. Although it might not be easy to explain the fact, yet we cannot doubt that the poison was the cause of this diseased state of the pylorus. Dr. Christison remarks, that in these cases of chronic poisoning, the pylorus is so contracted as scarcely to admit a probe. It would then appear that this is a very uniform morbid change. This circumstance should be borne in mind, since, in a case under criminal investigation, it might be urged by counsel, that the change should be ascribed to previous disease. As it is often extremely difficult to distinguish between the effect of poison and disease, in examining the dead body, so we cannot attach two strong a value to those cases in which the frequent recurrence of a peculiar morbid change like this, clearly indicates the cause to which it should be ascribed. Although *three months* may be regarded as a long period for an individual to survive the effects of a powerful mineral acid, yet a case is on record in which the patient did not die until after the lapse of *eight months.*]

*Henke's Zeitschrift*, 1836.

